# Antibodies against the SARS-CoV-2 S1-RBD cross-react with dengue virus and hinder dengue pathogenesis

**DOI:** 10.3389/fimmu.2022.941923

**Published:** 2022-08-15

**Authors:** Yi-Ling Cheng, Chiao-Hsuan Chao, Yen-Chung Lai, Kun-Han Hsieh, Jen-Ren Wang, Shu-Wen Wan, Hong-Jyun Huang, Yung-Chun Chuang, Woei-Jer Chuang, Trai-Ming Yeh

**Affiliations:** ^1^ Department of Medical Laboratory Science and Biotechnology, College of Medicine, National Cheng Kung University, Tainan, Taiwan; ^2^ Department of Microbiology and Immunology, College of Medicine, National Cheng Kung University, Tainan, Taiwan; ^3^ Leadgene Biomedical, Inc., Tainan, Taiwan; ^4^ Department of Biochemistry and Molecular Biology, College of Medicine, National Cheng Kung University, Tainan, Taiwan

**Keywords:** COVID-19, dengue virus, SARS-CoV-2, diagnosis, antibody-dependent enhancement

## Abstract

Severe acute respiratory syndrome coronavirus 2 (SARS-CoV-2) has spread globally since December 2019. Several studies reported that SARS-CoV-2 infections may produce false-positive reactions in dengue virus (DENV) serology tests and vice versa. However, it remains unclear whether SARS-CoV-2 and DENV cross-reactive antibodies provide cross-protection against each disease or promote disease severity. In this study, we confirmed that antibodies against the SARS-CoV-2 spike protein and its receptor-binding domain (S1-RBD) were significantly increased in dengue patients compared to normal controls. In addition, anti-S1-RBD IgG purified from S1-RBD hyperimmune rabbit sera could cross-react with both DENV envelope protein (E) and nonstructural protein 1 (NS1). The potential epitopes of DENV E and NS1 recognized by these antibodies were identified by a phage-displayed random peptide library. In addition, DENV infection and DENV NS1-induced endothelial hyperpermeability *in vitro* were inhibited in the presence of anti-S1-RBD IgG. Passive transfer anti-S1-RBD IgG into mice also reduced prolonged bleeding time and decreased NS1 seral level in DENV-infected mice. Lastly, COVID-19 patients’ sera showed neutralizing ability against dengue infection *in vitro*. Thus, our results suggest that the antigenic cross-reactivity between the SARS-CoV-2 S1-RBD and DENV can induce the production of anti-SARS-CoV-2 S1-RBD antibodies that cross-react with DENV which may hinder dengue pathogenesis.

## Introduction

In late December 2019, a novel coronavirus designated severe acute respiratory syndrome coronavirus 2 (SARS-CoV-2) spread rapidly worldwide, resulting in a global coronavirus disease 2019 (COVID-19) pandemic (1). SARS-CoV-2 is a positive-sense single-stranded RNA enveloped virus which is composed of at least four structural proteins: spike (S), envelope, membrane, and nucleocapsid (2). SARS-CoV-2 binds to the cell surface receptor angiotensin-converting enzyme 2 (ACE2) through trimeric S glycoprotein expressed on the viral envelope (2). Each monomer of the S protein is approximately 180 kDa and contains two subunits, S1 and S2. The receptor-binding domain (RBD) in the S1 subunit (S1-RBD) is an immunodominant region that is the main target of neutralizing antibodies (3–5). Symptoms of COVID-19 can be nonspecific, such as fever, cough, and tiredness, which may appear 2 to 14 days after exposure. Other symptoms can include shortness of breath or difficulty breathing, muscle aches, sore throat, headache, chest pain, and rash. However, in some patients, these symptoms can progress to life-threatening respiratory insufficiency and affect multiple organs, such as the heart, liver, and kidney (6). While the gold standard in COVID-19 diagnosis is reverse transcriptase polymerase chain reaction (RT–PCR), it requires complex sample manipulation and expensive machinery. To control the spread of SARS-CoV-2 and strengthen countries’ testing capacity, antigen and antibody rapid diagnostic kits are increasingly being used by many countries. However, several studies have reported that SARS-CoV-2 infections may produce false-positive antibody reactions in dengue virus (DENV) serology tests and vice versa, leading to misdiagnosis between COVID-19 and DENV infection based on rapid serological test results (7–10). Potential antigenic cross-reactivity between SARS-CoV-2 and DENV has been proposed to explain the false-positive serological test results among COVID-19 and dengue patients (11).

DENV infection, which is transmitted by Aedes mosquitoes, is prevalent in tropical and subtropical areas where the vector resides. However, it has dramatically increased in incidence within the last twenty years due to climate change and the convenient transportation system (12). It is estimated that greater than 2.5 billion people live in endemic areas, and the number of individuals infected by DENV is thought to exceed 50 million globally per year. DENV infection can cause mild dengue fever or more severe dengue hemorrhage fever (DHF) or dengue shock syndrome (DSS). DHF is a severe febrile disease characterized by abnormalities in homeostasis and increased capillary leakage that can progress to blood pressure decrease and hypovolemic shock (DSS) (13). However, most dengue patients show only flu-like illness, which is very similar to COVID-19. Therefore, the concurrence of SARS-CoV-2 and DENV infections has become a serious challenge for public health and medical management in dengue-endemic areas (14).

DENV is a positive-stranded RNA enveloped virus (15). It is composed of three structural proteins, namely, core protein (C), membrane-associated protein (M) produced as a precursor protein (prM), and envelope protein (E), and 7 nonstructural proteins (NSs). Based on the antigenic difference of the E protein, DENV can be divided into four different serotypes, DENV 1-4. Dengue NS1 is a glycosylated 48-kDa protein that can be secreted as a hexamer into the blood circulation during DENV infection. Circulating soluble NS1 can disrupt endothelial cell integrity and increase endothelial permeability (16–19). In addition, antibody-dependent enhancement (ADE) has been proposed to explain why many of the cases of DHF/DSS occur following secondary infection with a serotype of DENV different from that causing previous infection. Based on ADE, preexisting antibodies generated from previous infection or vaccination do not neutralize secondary infection of different serotypes, but enhance it, possibly by triggering Fc receptor-mediated virus uptake. Consequently, more severe disease may occur (20–22). ADE has been documented not only in DENV but also other respiratory virus infections, including SARS-CoV (20–22). Anti-SARS-CoV-2 antibodies could exacerbate COVID-19 through ADE has been suggested as well (23).

Previously, a computational simulation study revealed that a monoclonal antibody (mAb) against DENV E could bind to the SARS-CoV-2 S1-RBD and potentially block human ACE2 receptor binding (24). However, it remains unclear whether SARS-CoV-2 and DENV cross-reactive antibodies provide cross-protection against each disease or promote ADE and increase the risk of disease severity (14). To address this question, we first demonstrated that antibodies that cross-reacted with SARS-CoV-2 spike protein and the S1-RBD were increased in dengue patients’ sera. Furthermore, we purified anti-S1-RBD IgG from SARS-CoV-2 S1-RBD hyperimmune rabbit (Rbt) sera and found that it could cross-react with dengue E and NS1. In addition, anti-S1-RBD IgG could inhibit DENV infection and block NS1-induced endothelial hyperpermeability *in vitro* and prevent DENV-induced prolonged bleeding time and decreased NS1seral level in mice. Last, an increase in antibody binding to DENV-related antigens was also found in some individuals after SARS-CoV-2 infection. Furthermore, DENV infection *in vitro* was inhibited in the presence of COVID-19 patients’ but not healthy controls’ sera. Thus, our results suggest anti-SARS-CoV-2 antibodies induced during SARS-CoV-2 infection may interfere with DENV infection, which should be further evaluated in clinical study.

## Materials and methods

### Recombinant proteins and peptides

DENV serotype 2 E, prM and NS4B recombinant proteins were expressed and purified from *Escherichia coli* (Leadgene Biomedical Inc., Tainan, Taiwan). SARS-CoV-2 trimeric spike and nucleocapsid protein purified from HEK293 cells and human ACE2-Fc recombinant protein purified from CHO cells were provided by Leadgene company (Cat. No. 63233, 61633 and 63333). The synthetic peptides were customized, purified and synthesized by Leadgene company. DENV serotype 2 (strain Thailand/16681/84) NS1 recombinant protein produced in mammalian HEK293 cells was purchased from The Native Antigen Company (Oxfordshire, UK).

### Human serum

Dengue patient sera were collected from National Cheng Kung University Hospital (NCKUH) at the acute stage of the disease during a DENV outbreak in Tainan, Taiwan, in 2015 (25). In addition, sera from 29 healthy donors were included as negative controls. All serum collections were performed in accordance with the relevant guidelines and regulations approved by the institutional review board of NCKUH (IRB #A-BR-101–140). SARS-CoV-2 antibody positive sera were purchased from Access Biologicals (Vista, CA), and the detailed information was shown in the supplementary table of our previous study (26). According to the manufacturer’s documentation, the samples were collected in the U.S. in June 2020 from 30 COVID-19 patients (19 belong to the race of Caucasian, 10 African American, and one unknown) who were confirmed of infection during March to April 2020. The commercial COVID-19 patient sera were dispensed in a Biosafety Level-2 Plus (BSL-2+) laboratory by fully trained individuals according to the compliance policies of NCKUH. Dispensed patient sera were inactivated at 56°C for 30 min before being used in this study.

### Cell lines

The *Aedes albopictus* cell line (C6/36) and baby hamster kidney cell line (BHK-21), maintained in Dulbecco’s modified Eagle’s medium (DMEM) supplemented with 10% fetal bovine serum (FBS, HyClone, Logan, UT), were purchased from the American Type Culture Collection (ATCC, Manassas, VA) and Japanese Collection of Research Bioresources (Japan), respectively. The *Drosophila melanogaster* cell line (S2) purchased from ATCC was maintained in Schneider’s Drosophila Medium (SERVA Electrophoresis GmbH, Heidelberg, Germany) supplemented with 10% FBS. The human microvascular endothelial cell line (HMEC-1) was obtained from the Center for Disease Control and Prevention (CDC, Taiwan) and was cultured in Medium 200 (Thermo Fisher Scientific, Waltham, MA) supplemented with 10% FBS. Human monocytic cell line (THP-1) was grown in RPMI 1640 medium with 10% FBS. Except for S2 cells and C6/36 cells, which were cultured at 27 °C without CO_2_ incubation and at 27 ° C in a 5% CO_2_ atmosphere, respectively, the other cells were cultured at 37 °C in a 5% CO2 atmosphere.

### Viral stocks

The DENV serotype 2 strain 16681 or 454009A was propagated in C6/36 cells as previously described (27). To obtain high titers of DENV, we used a Macrosep Advance Centrifugal Device (MW cutoff of 30 kDa; Pall Corp., Port Washington, NY) to concentrate the DENV-containing medium *via* centrifugation at 6000×g at 4°C, and the concentrated DENV was stored below −70°C until use.

### Expression and purification of SARS-CoV-2 S1-RBD recombinant protein

For the expression and purification of SARS-CoV-2 S1-RBD recombinant protein, SARS-CoV-2 S1-RBD from a.a. 319 to 541 (YP_009724390) was cloned into pMT/BiP/V5-His B plasmid for the expression in S2 cells (**Supplementary Figure 1**). In brief, pMT-S1-RBD, containing 2x strep and 6x histidine, was transfected into S2 cells and induced with 500 µM CuSO_4_. After four days of induction, S1-RBD protein in the supernatant was purified by Strep-Tactin Superflow Plus (QIAGEN GmbH, Hilden, Germany). 2.6 mg of S1-RBD protein were purified from a 500 mL induction medium. The purity of SARS-CoV-2 S1-RBD from S2 cell was checked using SDS-PAGE and western blotting with anti-His antibody (Cat. No. 10411, Leadgene Biomedical Inc.). Human ACE2 binding ability of the purified SARS-CoV-2 S1-RBD was further confirmed by its binding to human ACE2-Fc recombinant protein by ELISA and colocalization with ACE2 in human ACE2 expressing Caco-2 cells using immunofluorescence confocal microscopy (data not shown).

### Immunization and antibody purification

For the preparation of anti-S1-RBD hyperimmune sera, two Rbt were primed and challenged on days 0, 14, and 28 with SARS-CoV-2 S1-RBD recombinant protein (250 µg for each Rbt) emulsified with incomplete Freund’s adjuvant (Sigma-Aldrich, St. Louis, MO). Sera were collected 7 days after the final immunization and stored at -20°C until use. The above immunization procedures were performed by Leadgene company. For the purification of Rbt IgG, the collected Rbt sera (50 mL) were heat inactivated at 56°C for 30 min. After fivefold dilution with PBS and filtering with a 0.45 µm syringe filter, IgG in the Rbt immune sera was purified by Pierce Protein G Plus Agarose (Thermo Fisher Scientific) and eluted with 0.1 M glycine-HCl (pH 2.7) and immediately neutralized with neutralizing buffer (1 M Tris-HCl, pH 9.0). The purified Rbt IgG was dialyzed against PBS at 4°C using SnakeSkin Dialysis Tubing with a 10 kDa molecular weight (MW) cutoff (Thermo Fisher Scientific). To obtain anti-S1-RBD IgG, S1-RBD affinity column was prepared by conjugation SARS-CoV-2 S1-RBD recombinant protein (5 mg) with 2 mL of NHS-activated Sepharose beads (Cytiva, Marlborough, MA) and blocked by ethanolamine followed the instruction provided by the manufacture. Purified Rbt IgG was incubated with S1-RBD-conjugated affinity column and eluted as described above to obtain anti-S1-RBD IgG. Those did not bind to SARS-CoV-2 S1-RBD affinity column after three rounds of incubation were also collected as flow-through Rbt IgG. In 50 mL of S1-RBD immunized rabbit serum, approximately 400 mg Rbt IgG could be purified from Protein G Plus Agarose. In addition, about 5.8 mg of anti-S1-RBD IgG, could be purified from 400 mg of these Rbt IgG by SARS-CoV-2 S1-RBD affinity column.

### Enzyme-linked immunosorbent assay

To investigate whether the antibodies could bind to the targeted proteins, indirect ELISA was performed. Briefly, 2 µg/mL proteins were coated onto a high-binding 96-well ELISA plate (50 µL/well) overnight at 4°C. After blocking with 1% BSA in PBS (200 µL/well), the samples (anti-S1-RBD IgG, anti-DENV NS1 mAb/pAb, or patient/healthy donor anti-sera) were serially diluted with 1% BSA and incubated in wells for 1 h at 37°C. The bound antibodies were detected with anti-Rbt/mouse IgG-horseradish peroxidase (HRP) antibodies (1:10,000) (Leadgene Biomedical Inc.) or anti-human IgG-HRP antibody (1:4000) (Thermo Fisher Scientific) (50 µL/well) for 1 h at 37°C. Wells were washed three times with PBST (PBS containing 0.01% Tween 20, 250 µL/well) between each step. For color development, TMB (50 µL/well) was added, the plates were incubated for 10-15 min, and the reaction was stopped by addition of 2N H_2_SO_4_ (50 µL/well). The absorbance was read at OD 450 nm by a VersaMax microplate reader (Molecular Devices, Sunnyvale, CA). To investigate whether a peptide could inhibit antibody binding to the targeted proteins, competitive ELISA was performed. Briefly, antibodies with the indicated concentration were preincubated with the serially diluted peptide in PBS with 1% BSA before incubating with protein-coated ELISA plate. The bound antibodies were detected with an anti-Rbt or ant-mouse IgG-HRP antibody. The color development was performed as described above.

### Western blotting

Trimeric spike protein and pseudovirus or concentrated supernatant of DENV were prepared under reducing condition prior to loading onto 10% SDS–PAGE gels for separation. The separated proteins were transferred onto a PVDF membrane (Pall, Ann Arbor, MI). The membrane was blocked with 5% skim milk in TBST (0.05% Tween 20 in Tris-buffered saline) and incubated with anti-S1-RBD IgG, anti-E poly IgG (Genetex), or anti-NS1 monoclonal IgG (33D2) overnight. To detect the bound IgG, the membrane was washed with TBST, followed by addition of a 1:10,000 dilution of HRP-conjugated anti-Rbt or anti-mouse immunoglobulin antibody (Leadgene). The bound HRP-conjugated antibodies were detected using WesternBright ECL (Advansta, San Jose, CA). The chemiluminescent signals were detected using an Image Quant LASS 4000 (GE Healthcare, Pittsburgh, PA).

### SARS-CoV-2 pseudovirus neutralization test

ACE2-overexpressing HEK293 cells (HEK293-ACE_O/E_) provided by Leadgene company were seeded on 96-well plates (3 × 10^4^cells/well) 18-24 h before infection. Antibodies with the indicated concentration were preincubated with 50 TCID_50_ of SARS-CoV-2 spike-expressing pseudovirus (lenti package with a nano luciferase reporter gene, which was kindly provided by Prof. Jen-Ren Wang’s laboratory) (28) for 1 h and added to the HEK293-ACE_O/E_ seeding plate. After 18-24 h, the infection rate was evaluated using a Nano-Glo Luciferase Assay System (Promega, Madison, WI), and the luciferase signal was detected by a SpectraMax iD5 (Molecular Devices).

### Immunofluorescence assay

C6/36 cells or DENV (strain 454009A)-infected C6/36 cells were seeded for 16-18 h and fixed with 4% paraformaldehyde for 15 min. Later, the cells were washed with PBS and blocked with SuperBlock™ Blocking Buffer (Thermo Fisher Scientific) for 1 h. Anti-S1-RBD IgG (1 µg/mL) and different anti-DENV mAbs such as anti-E mAb (50-2), anti-prM mAb (70-21), and anti-NS1 mAb (33D2) (1 µg/mL) were diluted with PBS and incubated with the cells overnight at 4°C. After being washed with PBS, the cells were incubated with Alexa 488-conjugated goat anti-mouse IgG (1:1000) and Alexa 594-conjugated goat anti-Rbt IgG (1:1000) (Invitrogen, Carlsbad, CA) in PBS for 1 h. Finally, the secondary antibodies were washed away, and the cells were mounted on the slide with DAPI Fluoromount-G mounting medium (Thermo Fisher Scientific). The prepared slides were visualized by inverted fluorescence microscope and FV3000-Confocal laser scanning microscope (Olympus, Japan).

### Epitope mapping using a phage-display random peptide library

To determine the epitopes recognized by anti-S1-RBD IgG, we used a phage-display random peptide library kit (PhD 12-mer; New England Biolabs, Ipswich, MA). Following the manufacturer’s suggestions, antibody (10 nM) was captured by protein A/G magnetic beads (Dynabeads; Invitrogen) for 30 min, followed by washing with 1 mL of Tris-buffered saline containing 0.5% Tween 20 (TBST). Phages (1 × 10^11^, 10 µL) from the original library were incubated with antibody complexes for 20 min, followed by washing 10 times with 1 mL of TBST. Negative selection with control Rbt IgG was performed at every round of panning. Unbound phages from negative selection were further incubated with anti-S1-RBD IgG complexes and washed as described above. Bound phages were eluted with glycine buffer (pH 2.2) and immediately neutralized using 1 M Tris-HCl (pH 9.0), followed by the amplification for subsequent rounds of panning. After three rounds of panning, the specific binding of positive single phage clones against anti-S1-RBD IgG was confirmed by sandwich ELISA using anti-S1-RBD IgG coated ELISA plates and an HRP-conjugated anti-M13 mouse mAb (Zymed Laboratories, South San Francisco, CA). The DNA sequences of isolated phages were analyzed using extracted single-stranded DNA (ssDNA) according to the manufacturer’s instructions.

### Fluorescent focus assay and focus reduction neutralization test

To determine DENV titer, BHK (1×10^4^/well) cells were seeded on 96 well plate for 16-18 h. Ten-fold serial dilutions of the virus stock or the sample containing DENV were added and incubated for 2 h at 37 °C (100 µL/well). For the FRNT, the antibodies (anti-S1-RBD IgG, control Rbt IgG, diluted COVID-19 patients’ or healthy donors’ sera) were preincubated with DENV serotype 2 strain 16681 (MOI=0.001) for 1 h before incubation. Later, the monolayers were overlaid with DMEM containing 2% FBS and 1% methylcellulose (100 µL/well), and the plates were incubated at 37 °C for another 4 days. The BHK cells were then fixed with 4% paraformaldehyde for 15 min at room temperature (RT). Later, the cells were washed with PBS. Virus foci were stained with an anti-NS1 antibody (mAb 33D2) (5 µg/mL) overnight at 4°C. After being washed with PBS, the cells were incubated with Alexa 488-conjugated goat anti-mouse IgG (Invitrogen) (1:1000) in PBS for 1 h. Finally, the secondary antibodies were washed away, and plaques were visualized using a DP72 fluorescence microscope (Olympus, Tokyo, Japan). The number of focus counted in the entire well was converted into the virus titer or infection rate (%).

### Antibody-dependent enhancement

To investigate whether anti-S1-RBD IgG could cause ADE during DENV infection, THP-1 cells were infected with DENV serotype 2 strain 16681 (MOI=10) with or without the indicated antibody. After 72 h, the supernatants were collected and viral titers were further calculated using FFA in BHK cells, as mentioned above.

### Transwell permeability assay

HMEC-1 cells (1 × 10^5^) were seeded on the upper chambers of Transwell plates (0.4 µm; Corning, The Netherlands) to form a monolayer. NS1 (2 µg/mL) was preincubated with the indicated concentrations of different antibodies for 1 h at 37°C before incubation with the HMEC-1 monolayer for another 24 h. To determine the permeability of the HMEC-1 monolayer, the upper chamber was reconstituted with 300 µL of serum-free medium, which contained 3 µL of streptavidin-HRP (R&D Systems, Minneapolis, MN). After 15 min, 50 µL of the medium in the lower chamber was transferred into a 96-well plate, and 50 µL of TMB substrate (R&D Systems) was added to the wells for color development. The reaction was stopped by addition of 50 µL of 2N H_2_SO_4_. The absorbance at 450 nm was measured by a VersaMax microplate reader.

### Mouse model of DENV infection

The animal study was performed in compliance with the Guide for the Care and Use of Laboratory Animals (The Chinese-Taipei Society of Laboratory Animal Sciences, 2010) and were approved by the Institutional Animal Care and Use Committee (IACUC) of NCKU under the number IACUC 109309. Six- to seven-week-old STAT1-deficient C57BL/6 (*STAT1-/-* B6) mice was used to infect DENV as previously described (29). The mice were maintained on standard laboratory food and water. To evaluate the protective effect provided by anti-S1-RBD IgG *in vivo*, *STAT1-/-* mice were intraperitoneally (i.p.) injected with mAb 33D2, control Rbt IgG, anti-S1-RBD IgG (150 µg/mouse) or PBS as a control 1 day before infection. Later, the mice were intravenously (i.v.) injected with concentrated DENV strain 16681 (1×10^7^ PFU/mouse) or concentrated C6/36 medium as a control. In addition, mAb 33D2, control Rbt IgG or anti-S1-RBD IgG (150 µg/mouse) was i.p. administered 24 h after DENV inoculation. Three days after DENV infection, the tail bleeding time was tested, and mice were sacrificed to determine NS1 level in the blood by quantitative NS1 ELISA.

### Bleeding time

Bleeding time was measured by cutting off 3–5 mm from the tip of the tail of the mouse. The duration of bleeding was recorded by monitoring the blood dripping onto filter paper every 30 s until the diameter of the blood droplet was smaller than 0.5 mm.

### NS1 quantitative ELISA

To quantify the NS1 levels, an in-house NS1 sandwich ELISA was performed. Briefly, 5 µg/mL anti-NS1 mAb 31B2 was coated onto 96-well plates at 4°C overnight. After blocking with 1% BSA in PBS for 1 h, mouse sera (1:4 dilutions) were coincubated with 2.5 µg/mL biotin-conjugated anti-NS1 mAb 33D2 at 37°C for 1 h. An HRP-labeled streptavidin solution (1:40) (R&D Systems) was added to the wells, which were incubated at RT for 40 min. After washing the wells three times with PBST (0.05% Tween 20 in PBS), TMB was added to the wells for color visualization. Following the addition of stop solution (2N H2SO4), the absorbance at 450 nm was read by a VersaMax microplate reader.

### Statistical analysis

All data were analyzed by GraphPad Prism version 5.0 (GraphPad Software Inc., CA). The *in vitro* and *in vivo* data are expressed as the means ± standard deviations (SDs) from three independent experiments. Student’s t test was used to analyze the differences between two groups One-way ANOVA with a Kruskal–Wallis comparison test was used to analyze the differences among multiple groups. P values <0.05 were considered statistically significant.

## Results

### Antibodies against the SARS-CoV-2 spike protein in archived dengue patient sera

**Figure 1:**
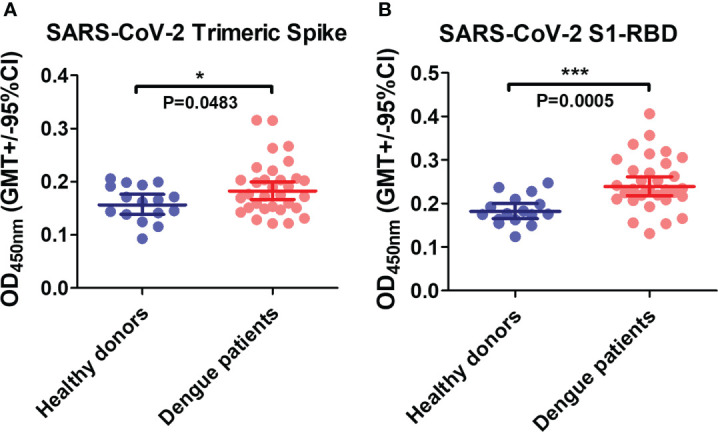
The presence of anti-SARS-CoV-2 antibodies in dengue patients’ sera. Recombinant SARS-CoV-2 proteins **(A)** trimeric spike and **(B)** S1-RBD were coated on ELISA plates. The bound antibodies were detected using an indirect ELISA. *P < 0.05, ***P < 0.001. (n = 32 for dengue patient; n=16 for healthy donor).

A Previous study revealed that the anti-DENV E mAb could bind to the SARS-CoV-2 S1-RBD in a computational simulation (24). Here, to investigate whether antibodies in dengue patient sera could cross-react with SARS-CoV-2 spike proteins, SARS-CoV-2 recombinant proteins (trimeric spike and S1-RBD) were coated on ELISA plates and bound IgG was detected. The results showed that dengue patient sera contain more antibodies that could cross-react with SARS-CoV-2 proteins than healthy donor sera. Furthermore, the binding of antibodies to SARS-CoV-2 S1-RBD was much significant increase than to the trimeric spike protein in dengue patients as compared with healthy donors (P=0.0005 vs. P=0.0483) (**Figure 1**).

### Anti-S1-RBD IgG purification and characterization

**Figure 2:**
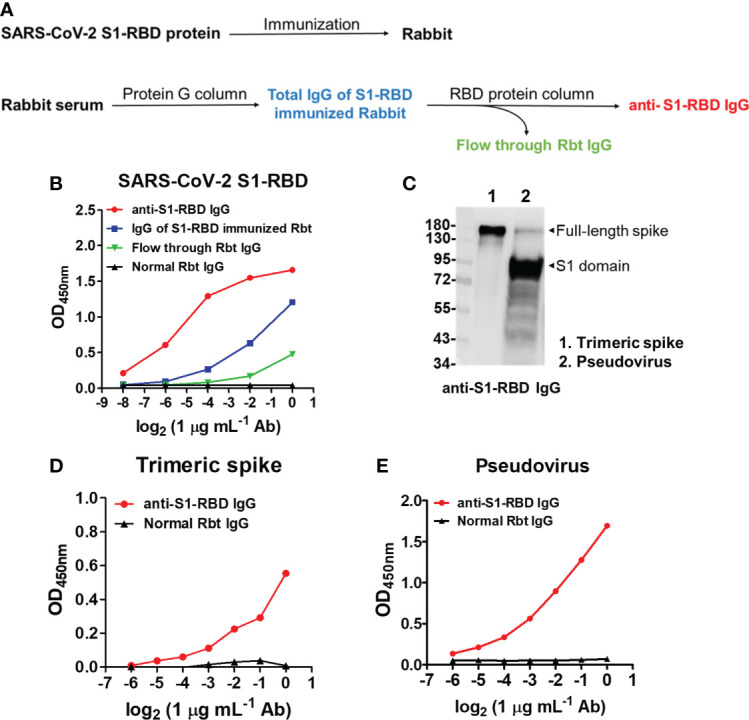
SARS-CoV-2 S1-RBD immunization and anti-S1-RBD IgG purification. **(A)** The flow chart of anti-S1-RBD IgG purification from anti-S1-RBD hyperimmune rabbit sera by protein G and RBD-conjugated affinity columns. **(B)** The binding ability of anti-S1-RBD IgG to the S1-RBD was detected by an indirect ELISA using an anti-Rbt IgG-HRP antibody. **(C)** The binding ability of anti-S1-RBD IgG to trimeric spike protein (lane 1) and SARS-CoV-2 pseudovirus (lane 2) was detected by Western blotting and by indirect ELISA **(D, E)**. The experiments were repeated two or three times with similar results, data from a single representative experiment was shown.

To further understand the antigenic cross-reactivity between the SARS-CoV-S1 RBD and DENV antigens, two Rbts were immunized with S1-RBD recombinant protein. Anti-S1-RBD IgG in the Rbt immune sera was purified by protein G agarose followed by S1-RBD-conjugated sepharose beads (**Figure 2A**). The binding ability of antibodies, including anti-S1-RBD IgG, IgG of S1-RBD-immunized Rbt serum, flow-through Rbt IgG, and normal Rbt IgG to the S1-RBD was compared using an indirect ELISA. The results showed that at the same concentration, the binding ability of anti-S1-RBD IgG was the highest, followed by IgG of S1-RBD-immunized Rbt serum, flow-through Rbt IgG, and normal rabbit IgG which showed no binding activity to the S1-RBD at all (**Figure 2B**). Since flow-through Rbt IgG was purified from the same immune Rbt sera, it was used as a control Rbt IgG (cRbt IgG) to compare with anti-S1-RBD IgG for later experiments. Next, to evaluate whether anti-S1-RBD IgG could recognized the RBD in native spike proteins, the binding ability of anti-S1-RBD IgG to the trimeric spike protein and a SARS-CoV-2 pseudovirus was evaluated using Western blotting analysis (**Figure 2C**) and indirect ELISA (**Figure 2D, E**). The results showed that anti-S1-RBD IgG could recognize both the trimeric spike proteins and the SARS-CoV-2 pseudoviruses in both denatured and native forms. In addition, anti-S1-RBD IgG also showed neutralization activity against SARS-CoV-2 pseudovirus infection in a dose-dependent manner with an IC50 (the half maximal inhibitory concentration) value of 50 µg/mL (**Supplementary Figure 2**).

### Anti-S1-RBD IgG cross-reacts with DENV proteins

**Figure 3:**
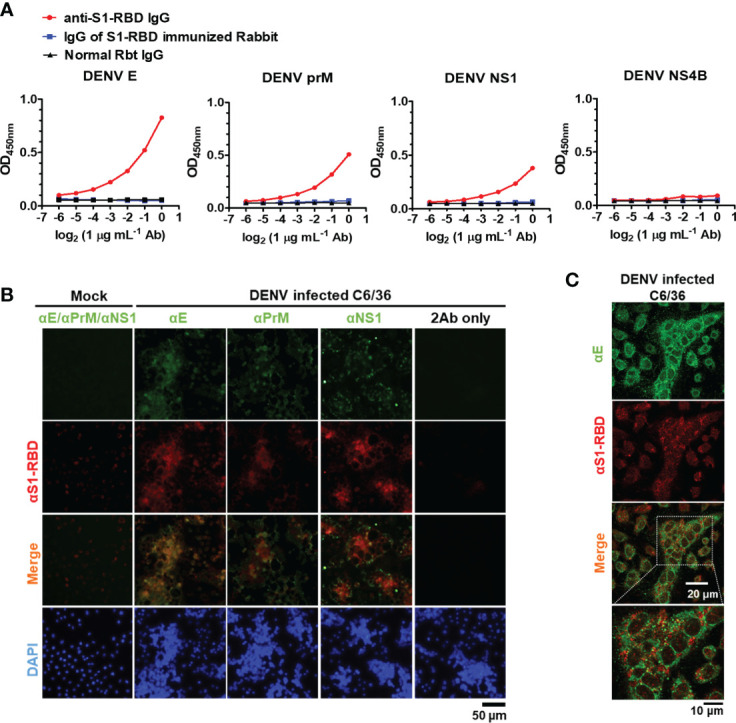
Anti-S1-RBD IgG cross-reacts with DENV proteins. **(A)** Recombinant DENV proteins, including envelope (E), precursor membrane (prM), and nonstructural proteins (NS1, NS4B), were coated in an ELISA plate. The cross-reactivity of anti-S1-RBD IgG to different DENV antigens was tested by an indirect ELISA. **(B)** The colocalization of anti-S1-RBD IgG (detected by goat-anti-Rbt-594) and different anti-DENV mAbs (detected by goat anti-mouse Alexa 488-conjugated antibodies) in DENV-infected C6/36 cells was determined by an immunofluorescent assay and visualized by Flourescence microscope DP72 (Olympus, Japan). **(C)** The colocalization of anti-S1-RBD IgG and anti-DENV E mAb were visualized by an FV3000-confocal laser scanning microscope (Olympus, Japan). The experiments were repeated two or three times with similar results, data from a single representative experiment was shown.

Next, to investigate whether anti-S1-RBD IgG could cross-react with DENV proteins, the cross-reactivity of IgG from S1-RBD-immunized Rbt serum and anti-S1-RBD IgG to different DENV proteins, including E, PrM, NS1, and NS4B was tested. We found that 1 µg/mL of anti-S1-RBD IgG could cross-react with DENV E, prM, and NS1, particularly DENV E, which had the strongest cross-reaction compared to that of the others (**Figure 3A**). However, no cross-reactivity to DENV NS4 was observed. Since all these recombinant proteins contained 6X His-tag, these results ruled out that the bindings of anti-S1-RBD IgG to DENV E, PrM, and NS1 were due to His-tag. Purified IgG from S1-RBD-immunized Rbt sera could also bind to DENV E, prM, and NS1, however, much higher doses were required as compared with what we found in anti-S1-RBD IgG (25 vs. 1 µg/mL) (**Figure 3A**; **Supplementary Figure 3**). In addition, the cross-reactions of anti-S1-RBD IgG to native DENV proteins were also confirmed in DENV-infected C6/36 cells. As shown in **Figure 3B**, anti-S1-RBD IgG could cross-react with DENV-infected C6/36 cells but not the C6/36 mock infection control cells. Similar staining patterns of anti-S1-RBD IgG and anti-DENV E was noticed. We, therefore, used immunofluorescence confocal microscopy to further visualize the colocalization of anti-S1-RBD IgG and DENV E as shown in **Figure 3C**. Moreover, the cross-reactions of anti-S1-RBD IgG to DENV antigens were also confirmed in the concentrated supernatant of DENV by western blotting (**Supplementary Figure 4**). The results showed that both E and NS1 proteins of DENV were recognized by anti-S1-RBD IgG and the band of E protein recognized by anti-S1-RBD IgG was much stronger than the band of NS1 protein. These results suggest that most of the anti-S1-RBD IgG recognized E protein.

### Identification of the sequences/epitopes recognized by Anti-S1-RBD IgG using a phage-displayed 12-mer random peptide library kit

To further identify the epitopes recognized by anti-S1-RBD IgG and investigate the antigenic similarity between the SARS-CoV-2 S1-RBD and DENV E, a phage-displayed 12-mer library kit was used. The detailed procedure was described in the materials and methods, and the workflow was presented in **Supplementary Figure 5A**. In brief, 10^11^ pfu of phages were negatively selected by flow-through Rbt IgG, followed by positive selection using anti-S1-RBD IgG. After three rounds of panning, fourteen single phage colonies were selected and the binding ability of these phages to anti-S1-RBD IgG were confirmed by a sandwich ELISA (**Supplementary Figure 5B**). The DNA of the selected phage was analyzed by Sanger sequencing and the nucleotide sequence encoding the displayed 12-mer peptide was further converted into the amino acid sequence. Twelve of fourteen (85.7%) phages showed an amino acid sequence of TQFEKASVNTTR (phage epitope 1), and two of fourteen (14.3%) showed an amino acid sequence of RDISIVPWNIRT (phage epitope 2) (**Supplementary Figure 5C**). This result suggested that most of the anti-S1-RBD IgG recognized the sequence of TQFEKASVNTTR (phage epitope 1).

### The peptide TQFEKASVNTTR competitively reduces Anti-S1-RBD IgG binding to the SARS-CoV-2 S1-RBD and DENV E protein

**Figure 4:**
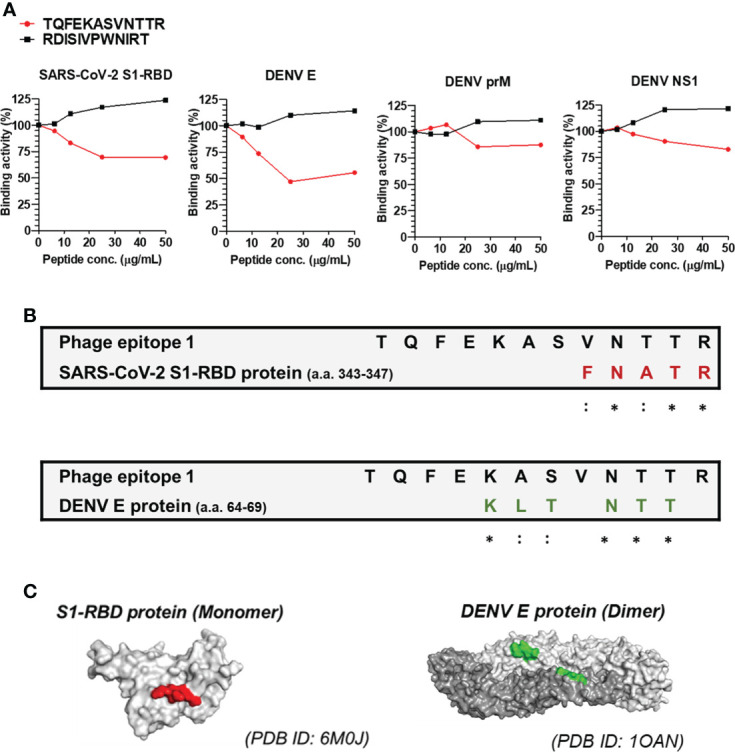
Epitope mapping of anti-S1-RBD IgG binding to DENV E protein. **(A)** Anti-S1-RBD IgG (0.125 μg/ml) was preincubated with a 2-fold dilution of synthetic free peptide of phage epitope 1 (TQFEKASVNTTR), and the binding ability to DENV proteins was detected by a competitive ELISA. **(B)** Alignment of the phage epitope 1 (TQFEKASVNTTR) with the SARS-CoV-2 S1-RBD and DENV envelope protein. *: identical,::conservative amino acid. **(C)** The positions of the consensus protein sequence in the structures of SARS-CoV-2 S1-RBD or DENV envelope protein are shown in red or green, respectively.

Since the peptide TQFEKASVNTTR was recognized by most of the anti-S1-RBD IgG, we further confirmed whether the peptide TQFEKASVNTTR contributed to the cross-reaction of anti-S1-RBD IgG to DENV E by the competitive ELISA (**Figure 4**). The results showed that the binding ability of anti-S1-RBD IgG to the SARS-CoV-2 S1-RBD and DENV E protein but not prM nor NS1 was significantly decreased by the TQFEKASVNTTR peptide in a dose-dependent manner (**Figure 4A**). On the other hand, the RDISIVPWNIRT peptide showed no inhibition on the binding of anti-S1-RBD IgG to the SARS-CoV-2 S1-RBD, DENV E, prM, or NS1 proteins. In addition, we also aligned the phage epitope (TQFEKASVNTTR) and SARS-CoV-2 S1-RBD protein sequence using BioEdit. The result showed that the sequence of amino acids (a.a.) 343-347 (FNATR) in the S1-RBD protein is similar to part of the sequence of phage epitope 1 (VNTTR) (**Figure 4B**). The amino acid positions 343-347 in the S1-RBD protein structure (PDB ID: 6M0J) was found to be located on the surface of the S1-RBD protein structure using PyMOL (**Figure 4C**). We further analyzed the epitope of DENV E protein recognized by anti-S1-RBD IgG using PyMOL visualization (DENV E protein structure, PDB ID: 1OAN) and sequence alignment by BioEdit (**Figure 4B**). The result suggested that anti-S1-RBD IgG might cross-react to DENV E protein a.a. 64-69, which are also located on the E protein surface (**Figures 4B, C**).

### Anti-S1-RBD IgG inhibits DENV infection and NS1-induced endothelial hyperpermeability without causing ADE *in vitro*

**Figure 5:**
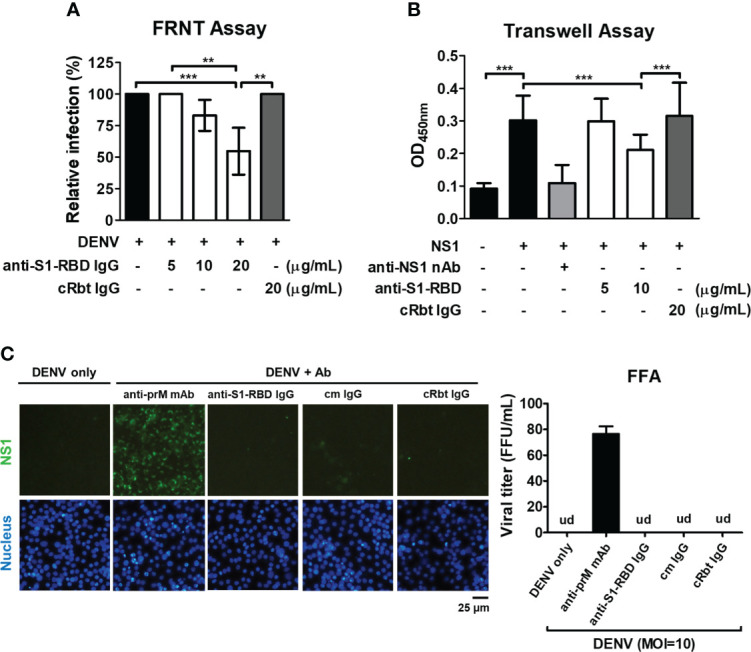
Influence of anti-S1-RBD IgG on DENV infection *in vitro*. **(A)** The neutralizing ability of different concentrations of anti-S1-RBD IgG or cRbt IgG against DENV infection was tested by a FRNT assay. **(B)** The permeability of HMEC-1 cells was measured by a Transwell assay. Different concentrations of antibodies as indicated were preincubated with DENV recombinant NS1 (2 µg/mL). The anti-NS1 mAb 33D2 (5 µg/mL) was used as a positive control. **(C)** ADE assay was performed in DENV infection (MOI=10) of THP-1 cells. Anti-S1-RBD IgG, an anti-prM mAb, control mouse (cm) IgG or control rabbit (cRbt) IgG (0.15 µg/mL) were incubated with DENV before infection. The infection of the cells was observed by immunofluorescent microscopy (left) and measured by FFA to determine viral titer (right). An anti-prM mAb (clone 70-21) was used as a positive control. Viral titer was un-detectable (ud) in all groups except in anti-prM mAb-treated group. **P < 0.01, ***P < 0.001.

To investigate whether anti-S1-RBD IgG could inhibit DENV infection, FRNT assay was used. The results showed that anti-S1-RBD IgG could decrease DENV infection *in vitro* in a dose-dependent manner. Significant inhibition of DENV infection was found when the concentration of anti-S1-RBD IgG reached to 20 µg/mL, while 20 µg/mL cRbt IgG did not (**Figure 5A**). In addition, since anti-S1-RBD IgG could bind to DENV NS1 (**Figure 3A**), a critical viral protein which could directly induce vascular leak (30–32), we investigated whether anti-S1-RBD IgG could block NS1-induced hyperpermeability in endothelial cells (HMEC-1) using Transwell assay. Surprisingly, the endothelial hyperpermeability induced by 2 µg/mL DENV NS1 could be blocked by 10 µg/mL anti-S1-RBD IgG, while 20 µg/mL cRbt IgG could not (**Figure 5B**). Anti-NS1 mAb 33D2, a homemade mAb (5 µg/mL) which can block NS1-induced hyperpermeability as previously described (33) was used as the positive control in this experiment. On the other hand, since ADE is a general concern in dengue infection, we further confirmed whether anti-S1-RBD IgG might cause ADE in DENV infection using human monocytic cell line THP-1 cells which express Fc receptor on their surface. Anti-prM mAb (clone 70-21) was used as a positive control for ADE (34). The result showed that DENV (MOI=10) could infect THP-1 cells only in the presence of anti-prM mAb (0.15-2.5 µg/mL). Twofold serial dilutions of anti-S1-RBD IgG, control mouse (cm) IgG, or cRbt IgG from 10 µg/mL were tested for their ability to enhance DENV infection of THP-1 cells. However, no enhancement of DENV infection in THP-1 cells was found in any concentrations of these antibodies we tested (**Figure 5C**).

### Possible epitope of DENV NS1 recognized by anti-S1-RBD IgG

**Figure 6:**
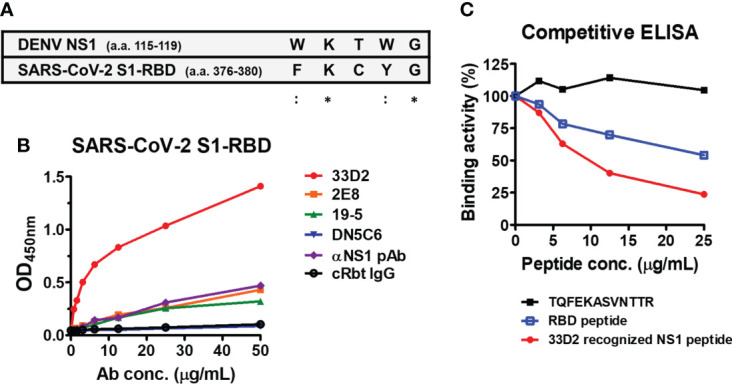
Monoclonal antibody against NS1 (33D2) cross-reacts with the SARS-CoV-2 S1-RBD. **(A)** Alignment between the epitope of DENV NS1 recognized by mAb 33D2 and SARS-CoV-2 S1-RBD protein sequence. *: identical;::conservative amino acid. **(B)** The binding of different NS1 antibodies to SARS-CoV-2 S1-RBD was measured by an indirect ELISA. **(C)** Competitive ELISA of mAb 33D2 binding to SARS-CoV-2 S1-RBD in the presence of different peptides. Anti-DENV NS1 mAb 33D2 (0.625 µg/ml) was preincubated with 2-fold dilutions of synthetic peptide (TQFEKASVNTTR), RBD peptide (VLYNSASFSTFKCYG), and 33D2-recognized NS1 peptide. The binding ability of the mAb 33D2 against the SARS-CoV-2 S1-RBD was detected by anti-mouse IgG-HRP antibodies. The experiments were repeated two or three times with similar results, data from a single experiment was presented.

Since anti-S1-RBD IgG, like anti-NS1 mAb 33D2, can block NS1-induced endothelial hyperpermeability and the epitope recognized by the mAb 33D2 is known (33). We compared the sequences recognized by the mAb 33D2 and the S1-RBD and found sequence homology between NS1 (a.a. 115-119) and the S1-RBD (a.a. 376-380) (**Figure 6A**). It was further observed that only the mAb 33D2 but not other anti-NS1 mAb (2E8, 19-5. DN5C6) (30) and anti-NS1 polyclonal antibodies (pAb) could cross-react with the SARS-CoV-2 S1-RBD (**Figure 6B**). Furthermore, the results from competitive ELISA showed that mAb 33D2 binding to the SARS-CoV-2 S1-RBD was blocked in the presence of 33D2 recognized NS1 peptide (a.a. 109-122 of NS1, TELKYSWKTWGKAK) or RBD peptide (a.a. 367-381 of the SARS-CoV-2 S1-RBD, VLYNSASFSTFKCYG) but not phage epitope 1 (TQFEKASVNTTR) in a dose-dependent manner (**Figure 6C**), indicating there is a potential antigenic similarity between DENV NS1 and SARS-CoV-2 S1-RBD which can be recognized by anti-S1-RBD IgG.

### Anti-S1-RBD IgG protects mice from DENV infection-induced prolonged bleeding time and decreases NS1 level in mouse sera

**Figure 7:**
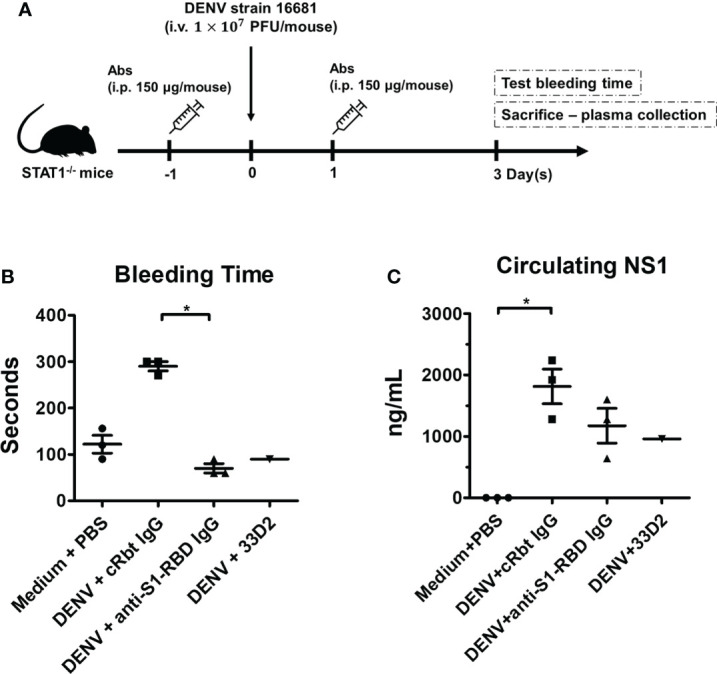
Anti-S1-RBD IgG protects mice from DENV infection-induced pathogenesis. **(A)** Anti-DENV NS1 (33D2), control Rbt IgG, and anti-S1-RBD IgG antibodies (150 µg/mouse) were intraperitoneally (i.p.) injected into STAT1-deficient C57BL/6 (STAT1-/- B6) mice, and one day later, 1 × 10^7^ pfu/mouse DENV was intravenously (i.v.) injected into the mice. On day one post-infection, the mice were i.p. injected with 150 µg of antibodies. **(B)** The tail bleeding time was tested on day 3 postinfection. **(C)** The NS1 level in the sera was determined by an NS1 quantitative ELISA [n = 3 for medium control, control Rbt IgG, and anti-S1-RBD IgG, n = 1 for anti-DENV NS1 antibody (33D2)]. *P < 0.05; Kruskal–Wallis ANOVA.

To evaluate the protective effect provided by anti-S1-RBD IgG against DENV infection *in vivo*, we evaluated DENV infection-induced hemorrhage in *STAT1*-/- mice (**Figure 7A**) (35). Mouse injected with the mAb 33D2, which can protect mice from DENV infection, was used as a positive control (34). In addition, mice injected with PBS or cRbt IgG were used as a negative control. The results showed that injection of mice with cRbt IgG 1 day before and 1 day after DENV infection induced bleeding time prolong and increased NS1 level in mouse sera as compared to those in mice injected with PBS without DENV infection. However, injection of mice with anti-S1-RBD IgG 1 day before and 1 day after DENV infection protected mice from DENV-induced prolonged bleeding time (**Figure 7B**) and reduced NS1 level in mouse sera as good as mouse injected with mAb 33D2 (**Figure 7C**). DENV titers in the sera of these mice after 3 days of infection were also evaluated by FFA using BHK cells; however, the DENV titers in these mice sera were too low to be detected.

### Antibodies against DENV in COVID-19 patients’ sera

**Figure 8:**
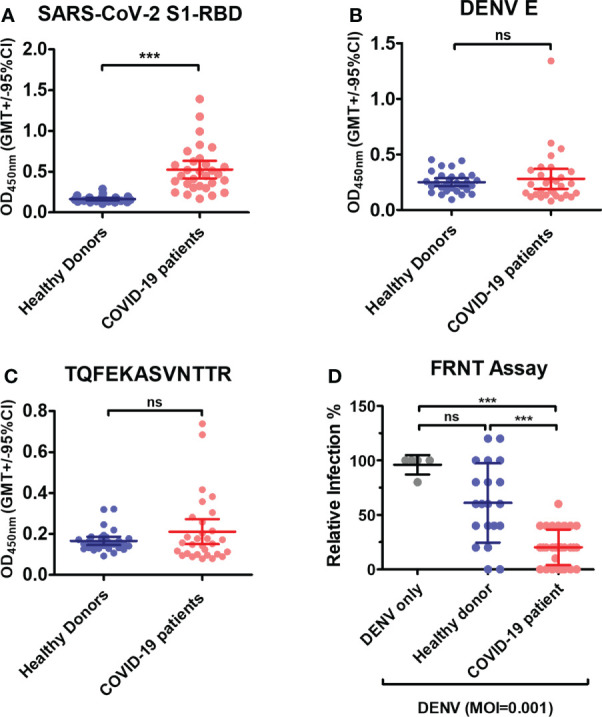
Antibodies in COVID-19 patients’ sera cross-react with DENV E and inhibit DENV infection *in vitro*. **(A)** Recombinant SARS-CoV-2 S1-RBD, **(B)** DENV E or **(C)** synthetic peptide of phage epitope 1 (2 µg/mL) was coated on ELISA plates. The binding of antibodies in COVID patient sera (1:100 diluted) to these plates was detected by an indirect ELISA. ***P < 0.001, ns indicates no significant difference between two groups as determined by t test. **(D)** The neutralizing ability of sera from COVID-19 patient (1:80 diluted) against DENV infection was tested by an FRNT assay. ***P < 0.001; Kruskal–Wallis ANOVA. (n = 30 for COVID-19 patient; n = 21 for healthy donor).

Lastly, the COVID-19 patients’ sera were used to investigate whether the cross-reaction of anti-S1-RBD IgG to DENV E protein can hinder DENV infection *in vitro*. As shown in (**Figure 8A**), the levels of antibodies against the SARS-CoV-2 S1-RBD recombinant protein were significantly increased in COVID-19 patients’ sera. The levels of antibodies binding to DENV E and the synthetic peptide of phage epitope 1 in COVID patients’ sera were also higher than those in healthy donors’ sera, even though, no statistical difference was found in COVID patients as compared to that in healthy donors (**Figure 8B, C**). To further investigate whether antibodies in COVID-19 patients’ sera could interfere with DENV infection, the neutralizing ability of sera from COVID-19 patient against DENV infection was tested by an FRNT assay. Surprisingly, the diluted (1:80) COVID-19 patients’ sera could significantly reduce DENV infection with a MOI of 0.001 in the FRNT system (**Figure 8D**). In fact, COVID patients’ sera with different dilution factors, from 1:20 to 1:160, showed neutralizing ability against dengue infection (data not shown). Furthermore, the neutralizing ability of sera from COVID-19 patients against DENV infection could be blocked in the presence of S1-RBD protein, but not SARS-CoV-2 nucleocapsid protein (**Supplementary Figure 6**). These results suggested that antibodies recognized S1-RBD may involve in the inhibition of DENV infection by COVID-19 patients’ sera.

## Discussion

In this study, we found a significant increase in antibody binding to SARS-CoV-2 trimeric spike and S1-RBD proteins from archived dengue sera collected from the 2015 dengue outbreak in Tainan city compared to that in healthy donor sera. Because these sera were collected predating the COVID-19 outbreak, we concluded that DENV infection may induce the production of SARS-CoV-2 cross-reactive antibodies and that most of these antibodies recognized the S1-RBD of spike protein. To further understand the antigenic cross-reactivity between the SARS-CoV-S1 RBD and DENV antigens, SARS-CoV-2 S1-RBD recombinant protein was used to immunize rabbits. Anti-S1-RBD IgG was purified from SARS-CoV-2 S1-RBD hyperimmune rabbit sera by both protein G and S1-RBD-conjugated affinity columns. These affinity-purified anti-S1-RBD IgG could recognize not only S1-RBD recombinant protein but also the trimeric spike protein and SARS-CoV-2 pseudovirus by ELISA and Western blotting analysis, indicating it could recognize S1-RBD in different native and denatured forms. In addition, anti-S1-RBD IgG could neutralize SARS-CoV-2 pseudovirus infection, even though the maximum inhibition was only about 50% at concentration of 50 µg/mL. Most importantly, these anti-S1-RBD IgG could cross-react with DENV recombinant proteins (including E, NS1, and PrM), concentrated dengue viral supernatant, and DENV-infected cells using different experimental approaches (including ELISA, western blotting, and IFA). Previously, antibodies in COVID-19 patient sera that can cross-react with DENV E and NS1 proteins have been reported (8, 11). In addition, possible similarities between SARS-CoV-2 epitopes in the HR2 domain of the S2 subunit of spike protein and the dengue E protein have been revealed by *in-silico* analysis (11). However, no experiment has been performed to prove that antigenic similarity between SARS-CoV-2 and DENV indeed can induce antibodies cross-react with each other. In this study, we are the first to demonstrate that immunization with SARS-CoV-2 S1-RBD indeed could induce DENV cross-reactive SARS-CoV-2 S1-RBD specific antibodies.

The results of dengue patients’ sera from this study also confirmed previous reports that archived dengue patient sera prior to the pandemic of 2019 SARS-CoV-2 contain antibodies which can cross-react with SARS-CoV-2. These SARS-CoV-2 cross-reactive antibodies in dengue patients can cause false-positive SARS-CoV-2 serology results (7, 36). Since the clinical and laboratory features of COVID-19 and dengue can be very similar, potential misdiagnosis between COVID-19 and dengue may arise if only rapid serological tests are used in dengue endemic regions (10, 37, 38). The misdiagnosis of both diseases will have serious consequences for both patient treatment and public health control. Therefore, in addition to antibody detection, antigen detection and RT–PCR to detect the viral genome should be performed to confirm the diagnosis.

Currently, the impact of antibody cross-reactivity with SARS-CoV-2 and DENV on the severity of both diseases in patients is still controversial. It is unclear whether antibodies induced by the antigenic cross-reactivity between DENV and SARS-CoV-2 can provide protective immunity overlap between these two diseases or worsen the disease through ADE. A recent study showed that previous SARS-CoV-2 infection may increase the risk of severe dengue (39). However, dengue fever might follow a less severe course, has also been found in children with recent SARS-CoV-2 infection (40). In these children, a trend toward a lower incidence of acute kidney injury and fewer organ dysfunctions has been reported. On the other hand, previous dengue infection may also increase the risk of severe COVID-19 due to cross-reactive non-neutralizing antibodies (41). To make the situation more complex, coinfection of SARS-CoV-2 and DENV is a significant public health problem, especially in dengue endemic areas. Therefore, the bidirectional impact of protection or ADE in COVID-19 and dengue is a growing concern (42, 43). In this study, we found that anti-S1-RBD IgG could inhibit DENV infection in BHK cells and DENV NS1-induced endothelial hyperpermeability in HMEC-1 cells. No ADE of DENV infection in THP-1 cells was found. Furthermore, these antibodies could reduce prolonged bleeding time and decrease NS1 seral level in DENV-infected mice. Therefore, our results suggest that SARS-CoV-2 S1-RBD-induced DENV cross-reactive antibodies may hinder dengue pathogenesis.

Four mAbs against DENV E that can neutralize DENV infection are predicted to bind to the S1-RBD, which is crucial for interaction with ACE2, based on *in silico* simulation (24). It is known that antibodies bound to E domain III (EDIII) are the most potent blockers of DENV (44). Another study also concluded that human mAbs recognizing EDIII, the EDI/EDII hinge, the E-dimer epitope, or a quaternary epitope involving EDI/EDII/EDIII are more potently neutralizing than antibodies recognizing the fusion loop (FL, 98–109 residues) of EDII (45). In this study, we used a phage-displayed random peptide library and identified that DENV E amino acid residues 64-69 were probably recognized by anti-S1-RBD IgG. Since anti-S1-RBD IgG could neutralize DENV infection only at low MOIs, residues 64-69 may represent a weak neutralizing epitope. It is known that N-linked glycans at Asn67 of DENV E dimer can bind to the carbohydrate recognition domain of DC-SIGN (46)., which are crucial for DENV binding and infection of cells (47). Therefore, we suspected that anti-S1-RBD IgG may interfere with the interaction between DENV E dimer and DC-SIGN, leading to the inhibition of DENV infection.

Based on the results of a competitive ELISA, the peptide TQFEKASVNTTR could inhibit only anti-S1-RBD IgG binding to the E protein, whereas it could not inhibit anti-S1-RBD IgG cross-reacting with prM or NS1. It is possible that the majority of the anti-S1-RBD IgG cross-reacted with DENV E. Therefore, the peptide (TQFEKASVNTTR) was identified by the phage-displayed random peptide library. Due to that, the epitope recognized by NS1 or prM cross-reactive anti-S1-RBD IgG could not be identified by this method. Since NS1 plays important roles in dengue pathogenesis (48, 49) and anti-S1-RBD IgG could also inhibit NS1-induced endothelial hyperpermeability, we tested the cross-reactivity of a few anti-NS1 mAbs with a SARS-CoV-2 S1-RBD protein-coated ELISA. Surprisingly, we found that only mAb 33D2 but not other anti-NS1 antibodies could cross-react with the SARS-CoV-2 S1-RBD. Since the epitope recognized by mAb 33D2 is known, we compared the NS1 a.a. 115-119 (WKTWG) sequence which was recognized by mAb 33D2 with SARS-CoV-2 S1-RBD protein and found a similar sequence FKCYG, which is located at a.a. 376-380 of SARS-CoV-2 S1-RBD. Thus, in addition to potential antigenic similarity between SARS-CoV-2 S1-RBD and DENV E protein, these results suggested that SARS-CoV-2 S1-RBD also contain potential antigenic similarity to DENV NS1 protein, which can induce antibodies that cross-react with NS1 and inhibit NS1-induced hyperpermeability.

In this study, even though we found significant increase of SARS-CoV-2 S1-RBD cross-reactive antibodies in the sera of dengue patients, the increase of DENV cross-reactive antibodies in the sera of COVID-19 patients was not significantly different from healthy controls. It is possible that the structural and conformational difference between SARS-CoV-2 S1-RBD and DENV (E and NS1) as well as the genetic background and immune status of different individuals may influence the generation of SARS-CoV-2 and DENV cross-reactive antibodies during DENV or SARS-CoV-2 infection. Nonetheless, we found DENV infection *in vitro* was inhibited in the presence of COVID-19 patients’ sera. This is consistent with a recent publication (50), in which the authors demonstrated that most of the sera of COVID-19 patients contain antibodies cross-react with DENV, which can neutralize dengue infection *in vitro*. Therefore, it is possible that DENV E protein cross-reactive anti-SARS-CoV-2 antibodies in COVID-19 patients’ sera may play a role in the inhibition of DENV infection. To further confirm this possibility, we pre-incubated the sera of COVID-19 patients with S1-RBD or nucleocapsid recombinant proteins in this study, and found that the effect of inhibiting dengue infection by COVID-19 patients’ sera could be reduced after preincubation with S1-RBD but not nucleocapsid protein. These results supported the phenomenon we observed in the experiments conducted with anti-S1-RBD IgG from rabbit serum and the hypothesis we proposed: anti-S1-RBD antibodies may inhibit dengue infection by cross-reacting with dengue antigens. However, we could not completely rule out the possibility of these COVID-19 patients having been infected by dengue previously or currently. Therefore, further investigation is needed to confirm the role of anti-S1RBD antibodies in inhibiting dengue infection. In summary, we demonstrated in this study that the antigenic similarity between the SARS-CoV-2 S1-RBD and DENV (E and NS1) has the potential to induce DENV cross-reactive antibodies after SARS-CoV-2 S1-RBD immunization or SARS-CoV-2 infection. These DENV cross-reactive antibodies may not only cause false-positive result in dengue serological test but also hinder dengue infection which should be further validated in clinical study.

## Data availability statement

The datasets presented in this study can be found in online repositories. The names of the repository/repositories and accession number(s) can be found in the article/**Supplementary Material**.

## Ethics statement

The studies involving human participants were reviewed and approved by the institutional review board (IRB). Dengue patient sera were collected from National Cheng Kung University Hospital (NCKUH) under the number IRB #A-BR-101–140. COVID-19-positive sera were purchased from Access Biologicals LLC. According to the manufacturer’s documentation, the samples were collected under IRB-approved protocols (SDP-001-FDA Licensed Plasmapheresis Center; SDP-002- U.S. Physician Network; SDP-003 Reference Laboratory Network). Written informed consent for participation was not required for this study in accordance with the national legislation and the institutional requirements. The animal study was reviewed and approved by the Institutional Animal Care and Use Committee (IACUC) of National Cheng Kung University (NCKU) under the number IACUC-109309.

## Author contributions

Y-LC, C-HC and T-MY conceived and designed the experiments. Y-LC, C-HC, K-HH and H-JH performed the experiments and analyzed the data. Y-LC, Y-CL, C-HC and T-MY wrote and edited the paper. J-RW, S-WW, Y-CC and W-JC provided comments and suggestions during the preparation of the manuscript. All authors contributed to the article and approved the submitted version.

## Funding

This study was supported by the Ministry of Science and Technology of Taiwan (MOST 110-2320-B-006-033, MOST 111-2321-B-006-009) and the National Health Research Institute (NHRI-110A1-MRCO-02212102).

## Acknowledgments

We appreciate the technical services provided by the “Bio-imaging Core Facility of the National Core Facility Program for Biotechnology, Ministry of Science and Technology, Taiwan, as well as the technical service provided by the Instrument Development Center of NCKU”.

## Conflict of interest

YC-C is employed by Leadgene Biomedical, Inc.

The remaining authors declare that the research was conducted in the absence of any commercial or financial relationship that could be considered a potential conflict of interest.

## Publisher’s note

All claims expressed in this article are solely those of the authors and do not necessarily represent those of their affiliated organizations, or those of the publisher, the editors and the reviewers. Any product that may be evaluated in this article, or claim that may be made by its manufacturer, is not guaranteed or endorsed by the publisher.
