# Supplementary material for: Antibodies against the SARS-CoV-2 S1-RBD cross-react with dengue virus and hinder dengue pathogenesis
Source: Front Immunol. 2022 Aug 15;13:941923. doi: 10.3389/fimmu.2022.941923 (PMC9420930; doi:10.3389/fimmu.2022.941923)
Supplement: Supplementary file 1 [file DataSheet_1.docx]

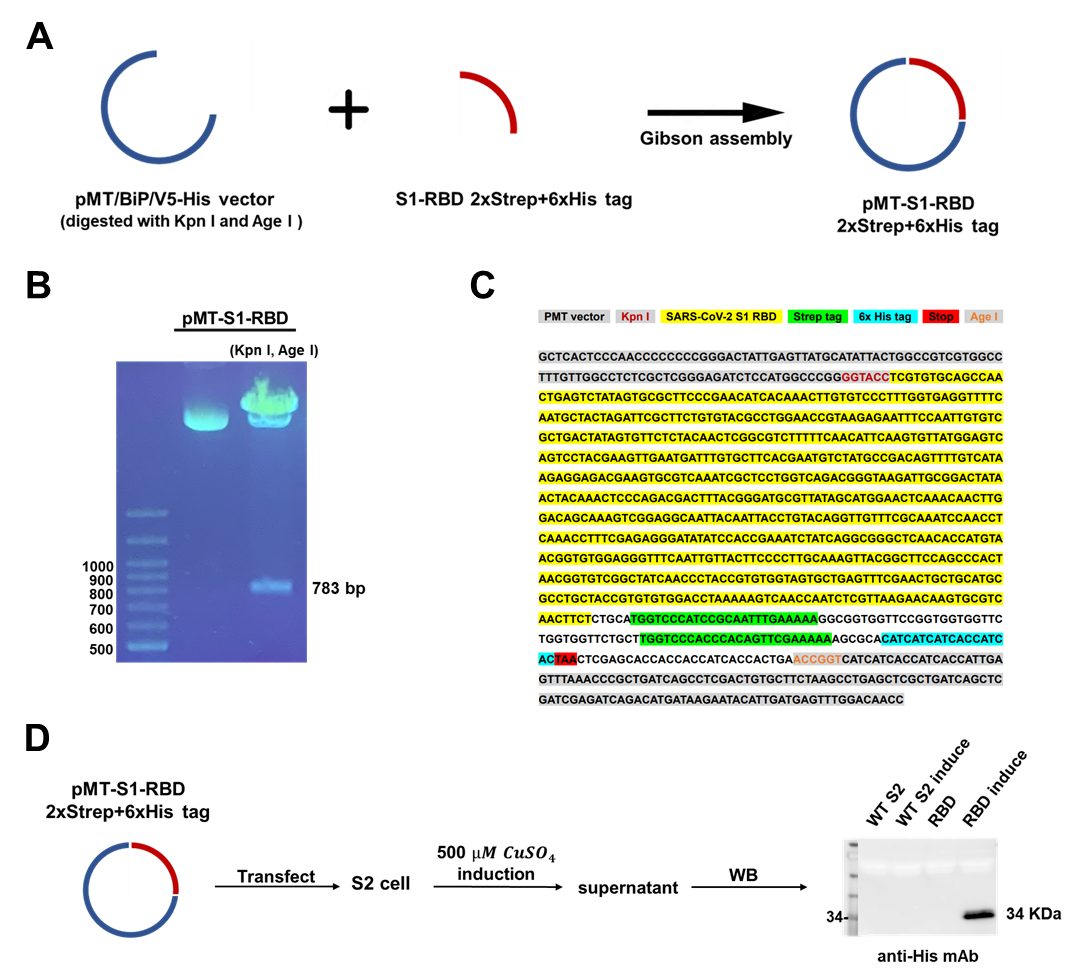


**Supplementary Figure 1. Construction and expression of the SARS-CoV-2 S1 RBD. (A)** The insert (SARS-CoV-2 S1-RBD, containing 2x streptavidin and 6x histidine tags) was synthesized and cloned into a pMT vector using a Gibson assembly kit. **(B)** The recombinant plasmid (pMT-S1-RBD) was digested by restriction enzymes (Kpn I and Age I) and confirmed by agarose gel electrophoresis. **(C)** The insert nucleotide sequence (S1-RBD) was verified by Sanger sequencing. **(D)** Flow chart of pMT-S1-RBD expression. Briefly, pMT-S1-RBD was transfected into S2 cells and induction with 500 μM CuSO_4_. S1-RBD protein expression in the supernatant of S2 cells was confirmed by Western blotting with anti-His antibody (lanes from left to right WT S2: wildtype S2 cells without pMT-S1-RBD transfection, WT S2 induce: WT S2 with CuSO4 induction, RBD: WT S2 with pMT-S1-RBD transfection, RBD induce: WT S2 with pMT-S1-RBD transfection and CuSO4 induction).


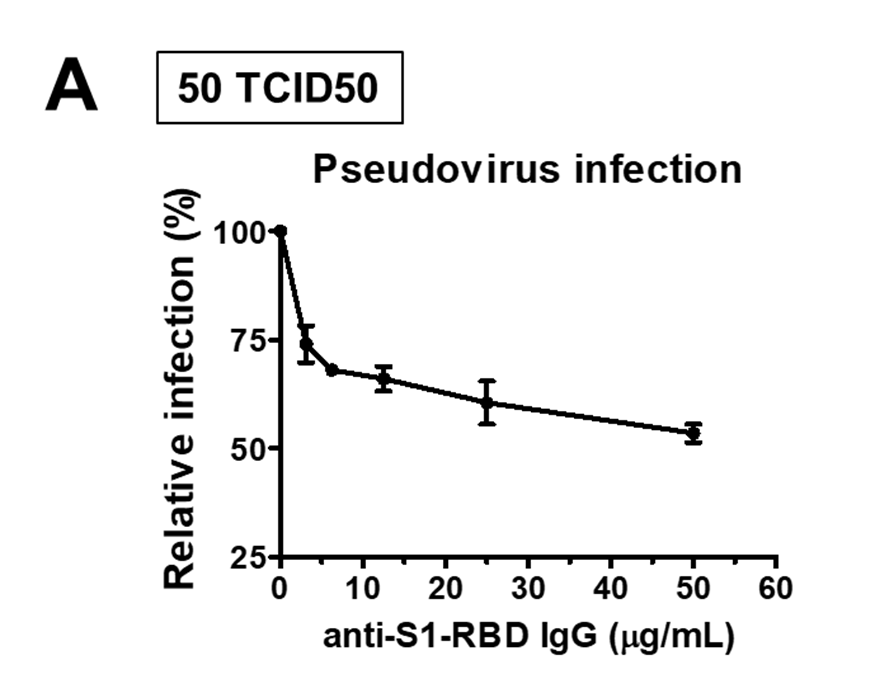


**Supplementary Figure 2. Anti-S1-RBD IgG inhibits SARS-CoV-2 pseudovirus infection.** The neutralization ability of anti-S1-RBD IgG was tested by pesudovirus neutralizing assay.

**
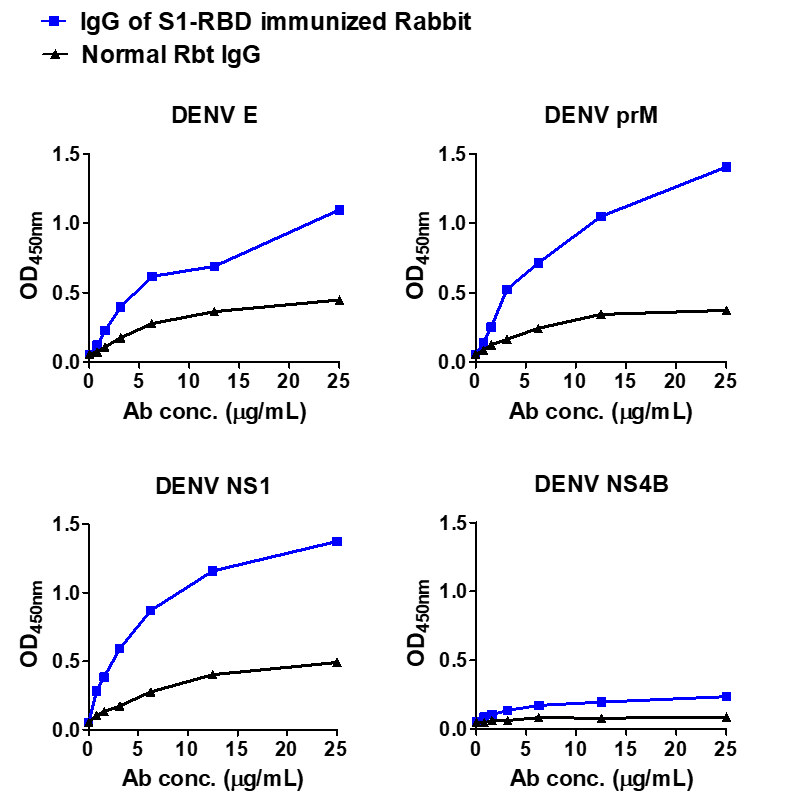
**

**Supplementary Figure 3. Purified IgG from S1-RBD immunized rabbit sera cross-reacts with DENV proteins.** Recombinant DENV proteins, including envelope (E), precursor membrane (prM), and nonstructural proteins (NS1, NS4B), were coated in an ELISA plate. The binding of purified IgG from S1-RBD immunized rabbit sera to different DENV antigens was detected by an indirect ELISA.


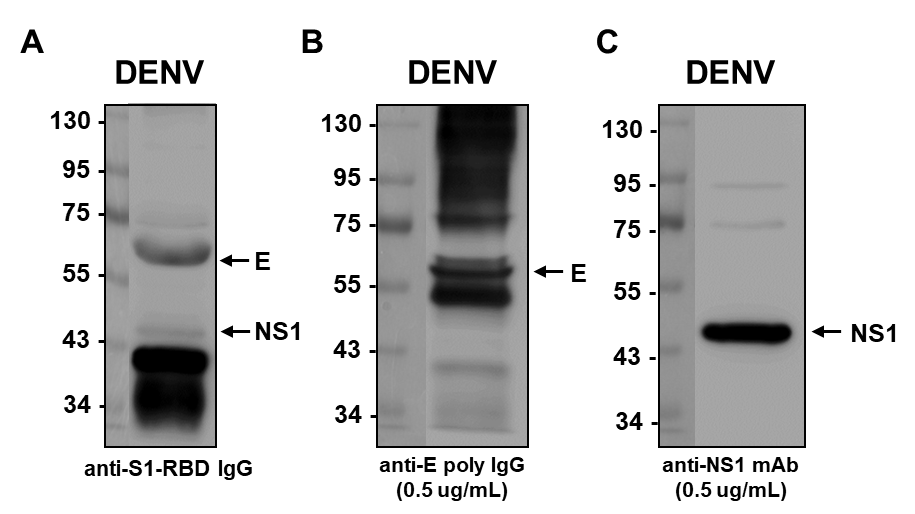


**Supplementary Figure 4. Anti-S1-RBD IgG cross-reacts with DENV E and NS1 proteins.** **(A)** The binding ability of anti-S1-RBD IgG to concentrated supernatant of DENV was detected by western blotting. The DENV antigens in the concentrated supernatant of DENV were confirmed by **(B)** anti-E poly IgG and **(C)** anti-NS1 mAb (33D2) (C).


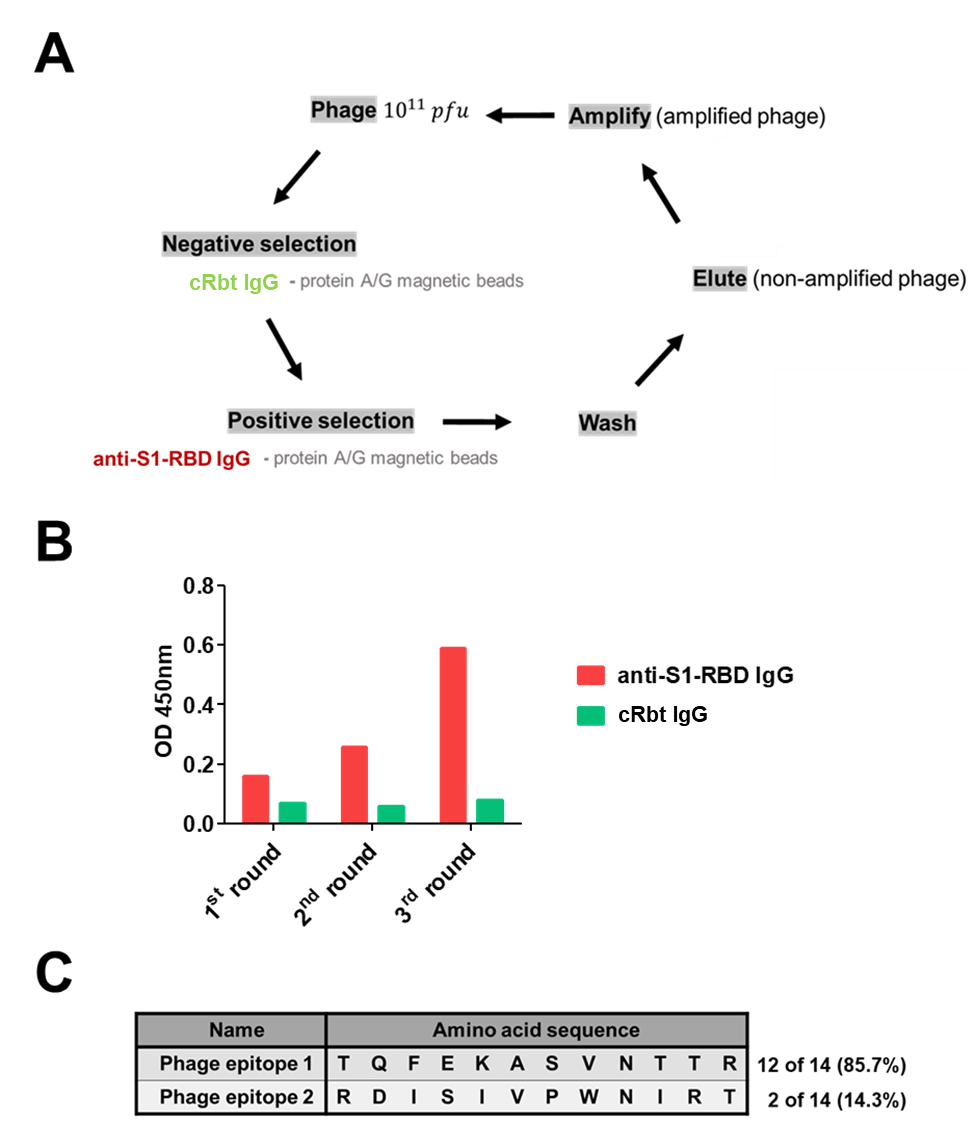


**Supplementary Figure 5.** **Identification of the sequences/epitopes recognized by anti-S1-RBD IgG using a phage-displayed 12-mer random peptide library kit. (A)** The flowchart of phage selection. Anti-S1-RBD IgG was preincubated with protein A/G magnetic beads for phage selection. The phage-displayed 12-mer peptide library was negatively selected by flow-through A/G magnetic beads with cRbt IgG, followed by positive selection using protein A/G magnetic beads with anti-S1-RBD IgG. Three rounds of panning were performed. **(B)** The selection of panning was confirmed by a sandwich ELISA. Anti-S1-RBD IgG/cRbt IgG was coated, and bound phages were detected using a HRP-conjugated anti-M13 antibody. **(C)** Fourteen single-colony phages were selected from the third-round titration plate. The phage DNA was purified and analyzed by Sanger sequencing, and the nucleotide sequence was converted into an amino acid sequence.


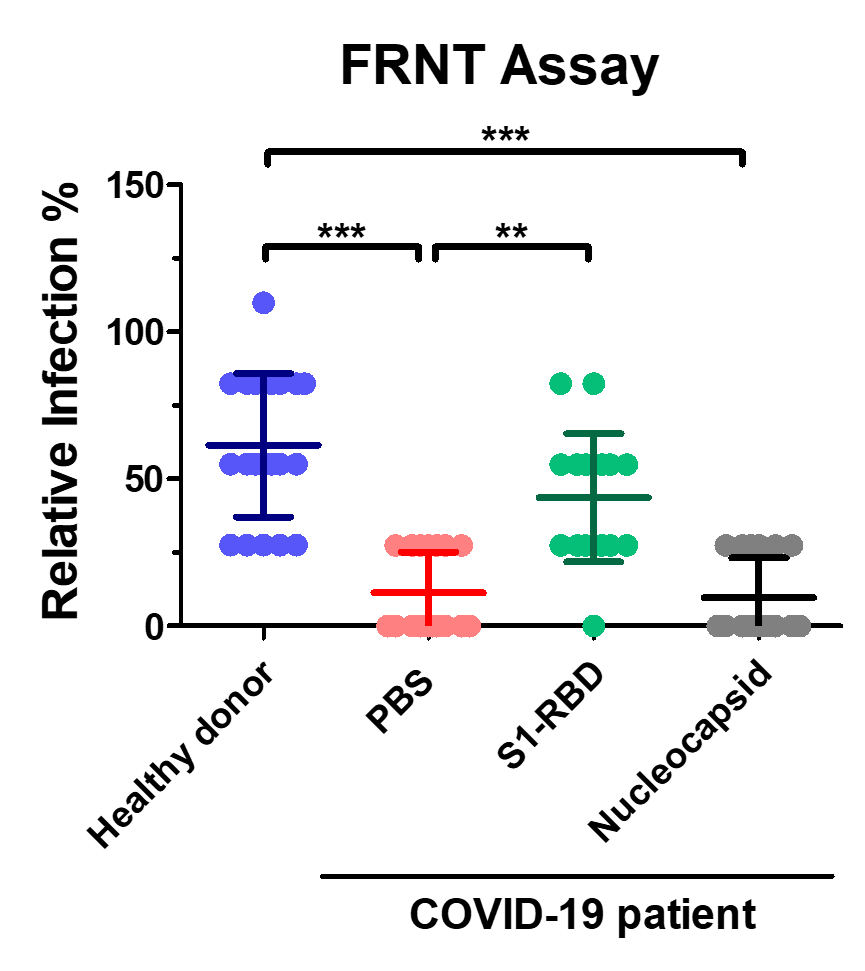


**Supplementary Figure 6.** **Anti-S1-RBD antibodies in COVID-19 patients’ sera inhibit DENV infection *in vitro*.** The sera (1:80 diluted) from COVID-19 patient were preincubated with S1-RBD protein (1 µg/mL) or nucleocapsid protein (1 µg/mL). The neutralizing ability of the sera against DENV infection were tested by an FRNT assay. **P<0.01, ***P < 0.001; Kruskal–Wallis ANOVA. (n = 17 for COVID-19 patient; n=21 for healthy donor)
